# Temporal trend analysis of the HIV/AIDS burden before and after the implementation of antiretroviral therapy at the population level from 1990 to 2020 in Ghana

**DOI:** 10.1186/s12889-023-16321-3

**Published:** 2023-07-20

**Authors:** Michael Boah, Daudi Yeboah, Mary Rachael Kpordoxah, Abdul-Nasir Issah, Martin Nyaaba Adokiya

**Affiliations:** 1 Department of Epidemiology, Biostatistics, and Disease Control, School of Public Health, University for Development Studies, Tamale, Ghana; 2 Department of Global and International Health, School of Public Health, University for Development Studies, Tamale, Ghana; 3 Department of Health Services, Planning, Management, and Economics, School of Public Health, University for Development Studies, Policy, Tamale, Ghana

**Keywords:** Antiretroviral therapy, HIV, AIDS, Incidence, Mortality, Ghana

## Abstract

**Background:**

Antiretroviral therapy (ART) has helped reduce the burden of human immunodeficiency virus (HIV) and acquired immune deficiency syndrome (AIDS) in the majority of countries. Its contribution to the HIV/AIDS burden in Ghana is still understudied. This study examined HIV/AIDS trends in Ghana before (1990–2004) and after (2004–2020) the implementation and expansion of ART.

**Methods:**

We obtained HIV/AIDS epidemiology and treatment data for the years 1990–2020 from the United Nations Programme on HIV/AIDS. We investigated the impact of the ART rollout on HIV/AIDS in Ghana using Joinpoint regression models.

**Results:**

The HIV incidence, prevalence, and AIDS-related deaths decreased significantly after 2004, as ART coverage increased from 1% to 2004 to 60% in 2020. The HIV incidence decreased by approximately 3% (AAPC = -2.6%; 95% CI: -3.2, -1.9) per year from 1990 to 2004 and approximately 5% (AAPC = -4.5%; 95% CI: -4.9, -4.2) per year from 2004 to 2020. Between 1990 and 2004, the HIV prevalence increased by approximately 5% (AAPC = 4.7%; 95% CI: 3.6, 5.8) per year but decreased by 2% (AAPC = -1.9%; 95% CI: -2.1, -1.6) per year between 2004 and 2020. Between 1990 and 2004, the annual increase in AIDS-related mortality was 14% (AAPC = 13.8%; 95% CI: 12.6, 15.0), but between 2004 and 2020, it decreased at nearly a 4% (AAPC= -3.6%; 95% CI: -4.7, -2.5) annual rate.

**Conclusions:**

We found trends indicating progress in Ghana’s fight against HIV/AIDS. However, the most significant declines occurred after the introduction of ART, suggesting that the scale-up of ART may have contributed to the decline in HIV/AIDS in Ghana. We advocate for the rapid expansion of ART in Ghana.

**Supplementary Information:**

The online version contains supplementary material available at 10.1186/s12889-023-16321-3.

## Background

In 2001, the United Nations held a special general assembly and agreed that the human immunodeficiency virus (HIV) and acquired immune deficiency syndrome (AIDS) were a global public health crisis [1]. In addition, it was decided to intensify international action and mobilise resources to combat the pandemic. Since 2000, unprecedented development assistance for HIV/AIDS has been provided. It has been established that more than $109.8 billion has been provided for HIV/AIDS in development assistance [2]. However, even after two decades, HIV and AIDS remain a major global health tragedy. Globally, at the end of 2020 alone, there were 1.5 million new infections of HIV, resulting in about 38 million people living with HIV (PLHIV), out of which 27.5 million were on HIV treatment [3]. There were 680,000 deaths from AIDS-related causes that occurred in the same year.

The availability of antiretroviral therapy (ART) combinations has transformed HIV/AIDS from a fatal to a chronic disease. ART is effective at preventing HIV in high prevalence settings, even in the presence of high levels of drug resistance and risky sexual behaviour, according to mathematical modeling [4, 5]. By suppressing viral load, ART reduces infectivity [6, 7]. As a result, individual morbidity, mortality, and onward transmission are reduced [8–12]. However, the benefits of ART are more pronounced when treatment is initiated early in the course of the disease and patients are able to adhere to therapy for the rest of their lives [8, 13, 14]. Furthermore, ART only makes HIV-infected people live longer; it does not result in viral eradication within individuals, and so it does not cure them [13, 14].

The first case of AIDS was reported in 1986 in Ghana, and the number of cases has since risen. According to the United Nations Programme on HIV/AIDS (UNAIDS), in 2020 there were 19,000 new HIV infections, resulting in national prevalence of 1.7% among adults and an estimated 350,000 PLHIV in Ghana [3]. In the same year, AIDS claimed the lives of 13,000 people. Priority interventions in Ghana’s response to the HIV/AIDS epidemic include advocacy, communication, and social mobilization to promote safer sex; management of sexually transmitted infections (STIs); safe blood; elimination of mother-to-child transmission; HIV testing and counseling; and addressing HIV/AIDS-related stigma and human rights abuse [15]. These interventions are geared towards reducing the number of new infections in the Ghanaian population. Ghana’s Prevention of Mother-to-Child Transmission (PMTCT) programme began with two facilities in the Manya Krobo District of the Eastern Region offering PMTCT services in 2002, while ART for the general population was introduced in June 2003 in the two pilot sites[16]. With funding from the Global Fund to fight AIDS, Tuberculosis, and Malaria, the government of Ghana rapidly expanded HIV counseling, testing, and treatment services to the rest of the country in 2005. The number of treatment sites increased from 2 to 2003 to 175 in 2013 and 197 at the end of 2015, providing ART to over 100,000 people. In September 2016, the Ghanaian government adopted the World Health Organization (WHO) policy of “Treat-All,“ which entails providing ART to all PLHIV regardless of their CD4 count, which was previously used as a cut-off point for treatment initiation [17]. After more than five years of use, coverage is still low. According to data from UNAIDS, only 60% of people in Ghana who required effective treatment were put on ART in 2020 [3]. Low levels of knowledge and misconceptions about HIV and AIDS, gaps in the quality of HIV/AIDS care, discrimination and stigmatization of PLHIV, and low use of HIV testing services are all big problems for Ghana in its fight against the HIV/AIDS epidemic [18–23].

In Ghana, extensive research on HIV and AIDS has been conducted. While most of the studies have focused on ART, such as level of utilization, adherence, and adverse drug reactions [18, 24–29], as well as HIV/AIDS knowledge and drug therapy [19, 20, 30–32], few have investigated HIV testing [22, 23], virological suppression [6, 7], or contraceptive use among HIV patients receiving chemotherapy [33]. However, we were unable to identify any study that investigated the trend of the HIV/AIDS burden in Ghana following the implementation of ART at the population level. Consequently, little is known about the HIV and AIDS burden in Ghana after the implementation of ART at the population level. More specifically, little attention has been given to the role of ART in the HIV/AIDS epidemic in Ghana.

The aim of the current study was to investigate the impact of ART rollout on the HIV/AIDS burden in Ghana by comparing the HIV/AIDS trends before and after ART implementation at the population level.

## Methods

### Data and sources

The HIV and AIDS annual epidemiological estimates were obtained from UNAIDS (https://aidsinfo.unaids.org/), which leads the world’s most extensive data collection on HIV epidemiology, programme coverage, and finance and publishes the most authoritative and up-to-date information on the HIV epidemic. Adult (15–49 years) HIV incidence (per 1000 uninfected adults 15–49 years), all-age incidence rate (per 1000 uninfected population), adult (15–49 years) HIV prevalence, number of PLHIV, AIDS-related deaths among children (0–14 years) and adults (15 + years), and AIDS-related deaths among all ages, were all collected in Ghana. In addition, data on ART coverage was gathered in Ghana. Unless otherwise specified, data were collected from 1990 to 2020.

### Statistical analysis

The temporal analyses were carried out using a joinpoint regression model. The model identifies the best line of fit across multiple years of data and employs an algorithm for testing whether a multi-segmented line is statistically superior to a straight line in describing the temporal trend of a dataset (Kim et al., 2000). The model assumes that the rate of change is constant over each joinpoint segment but varies across time segments. The significance test makes use of the Monte Carlo permutation method (i.e., it determines the “best fit” line for each segment). The analysis has two benefits. First, the model determines whether the indicator trend is upward, downward, or stationary. In other words, it identifies the years when significant changes occurred (joinpoint), allowing the magnitude of the change in each linear slope to be estimated by calculating the annual percentage change (APC). Second, once the line segments are established, the average annual percentage change (AAPC) over the entire period can be estimated. The Joinpoint Regression Program, version 4.9.1.0 April 2022 (Statistical Research and Applications Branch, National Cancer Institute, Calverton, MD), was used to conduct the analysis. The selection of joinpoints in this regression analysis was determined using the permutation test, a Monte Carlo resampling method. The range of joinpoints was defined from 0 to 5. Starting with the minimum of 0 joinpoints, we systematically tested whether additional joinpoints were statistically significant and should be included in the model, up to the maximum allowed. We used uncorrelated errors based on the assumption that there is no temporal dependence in the data. In other words, the data points are independent and the values at one time point do not influence or predict the values at subsequent time points. Statistical significance was set a *p* < 0.05. The following regression model was used to calculate the APC:

1 $$\text{log}\left({Y}_{x}\right)={b}_{0}+ {b}_{1}x$$

Where $$\text{log}\left({Y}_{x}\right)$$ is the natural log of the rate of the outcome in year ; $$x$$ is the year (e.g. 1990); and $${b}_{0} \text{a}\text{n}\text{d}$$$${b}_{1}$$ denote the intercept and slope of the segment (joinpoint), respectively.

The APC for a segment from year $x$ to year$x+1$ is given by:

2 $$APC=\frac{{e}^{{b}_{0}+{b}_{1}\left(x+1\right)}-{e}^{{b}_{0}+{b}_{1 }x}}{{e}^{{b}_{0}+{b}_{1 }x}} \times 100=\left({e}^{{b}_{1}}-1\right)\times 100$$

When there is no change in the trend, the APC remains constant and equals the AAPC. Otherwise, the points with trend changes divide the entire period (see Additional file 1). Then, the AAPC is calculated as a weighted average of the estimated APC in each segment, where the lengths of the segments are used as weights. We compared the change in various HIV indicators from 1990 to 2004, when population coverage of ART was 0% in Ghana, to the change from 2004 to 2020, when ART was implemented and at least 1% coverage was achieved.

Ethical considerations.

The data used in this study are freely available to any researcher from the UNAIDS database (https://aidsinfo.unaids.org/). This study relied on de-identified, publicly accessible secondary data, and so no ethical approval was required from a review board.

## Results

### Descriptive statistics

Table 1 shows the distribution of the variables studied in this study. From 1990 to 2020, the mean prevalence of HIV among adults in Ghana ranged from 1.2 to 2.5%. Adult HIV incidence ranged from 0.63 to 2.32 per 1000 uninfected population during the same period, with a mean incidence of 1.4. AIDS-related deaths (all ages) averaged 16,587.1, with a range of 3,900 to 24,000 deaths. ART coverage ranged from 0% to 2000 to 60% in 2020, with a mean coverage of 16.7%.

**Table 1:** Descriptive statistics of the variables investigated in this study (1990–2020)

Variable	n	Mean(SD)	Range
Adult (15–49 years) prevalence (%)	31	2.05(0.33)	1.2–2.5
Adult (15–49 years) incidence	31	2.11(0.95)	0.94–3.88
Incidence (All ages)	31	1.39(0.54)	0.63–2.32
Number of PLHIV (all ages)	31	276645.20(66208.53)	96,000–350,000
Coverage of ART (%) 2000–2020	21	16.71(16.81)	0–60
AIDS-related deaths (Children)	31	4300.00(1085.97)	1900–5700
AIDS-related deaths (Adults)	31	12206.45(4694.46)	2000–18,000
AIDS-related deaths (All ages)	31	16587.10(5637.60)	3900–24,000

AIDS: acquired immune deficiency syndrome; ART: antiretroviral therapy; PLHIV people living with HIV; SD: standard deviation

### Trend analysis of the incidence, prevalence, and number of people living with HIV in Ghana between 1990 and 2020

**Fig. 1:**
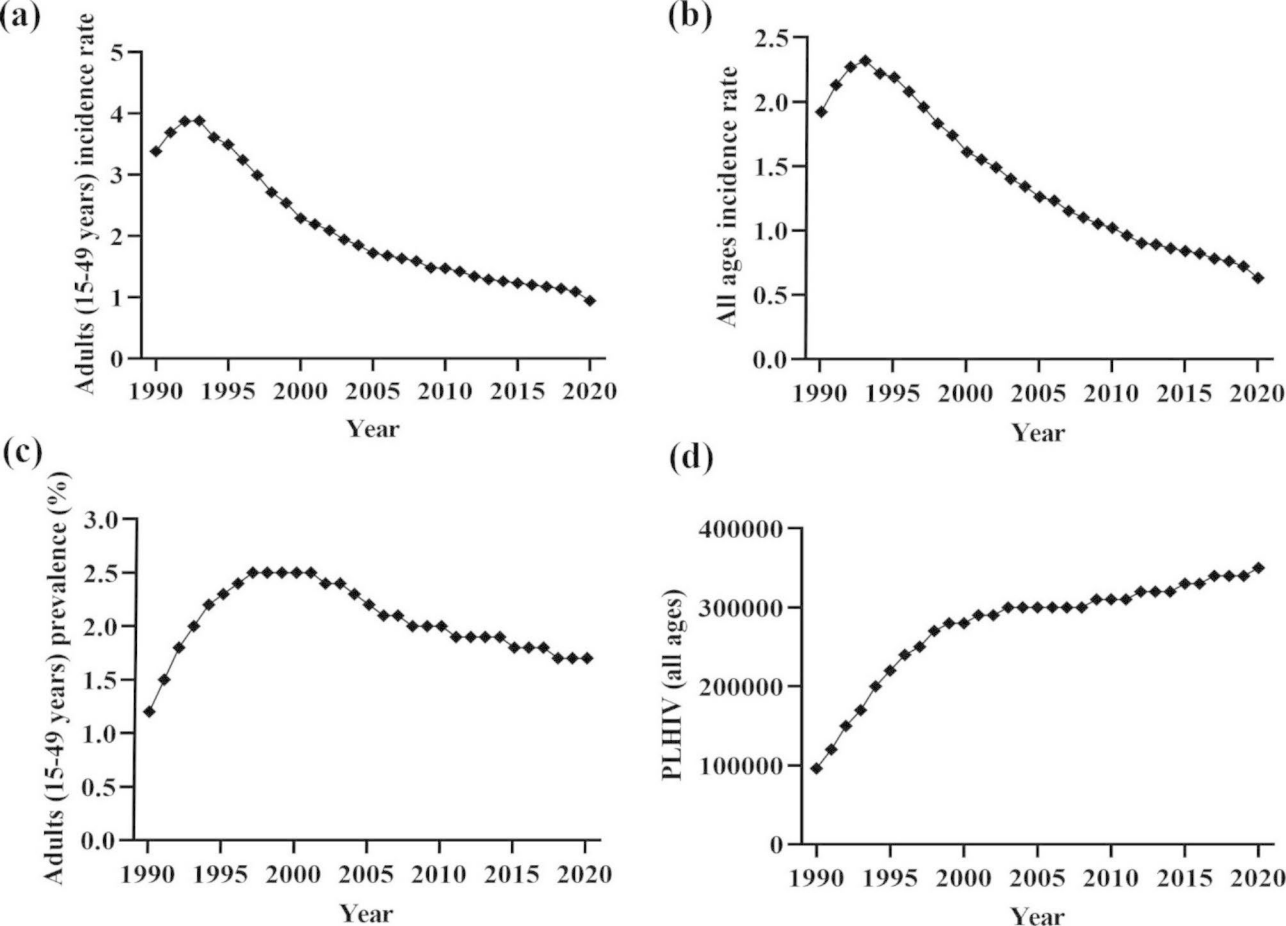
Trend analysis of the: **(a)** adult incidence rate (per 1000 uninfected population); **(b)** all age incidence rate (per 1000uninfected population); **(c)** adult (15–49 years) prevalence rate, and; **(d)** total number of people living with HIV (PLHIV) in Ghana between 1990 and 2020

The analysis of temporal trends shows that the HIV incidence rate in Ghana decreased between 1990 and 2020 among people of all ages and among adults aged 15 to 49. The declines, however, began in 1992 and 1993, respectively. Figure 1a shows that the adult incidence rate of HIV increased significantly at a rate of 4.5% (95% CI: 1.9, 7.2; *p* = 0.002) annually between 1990 and 1993 and then declined between 1993 and 2020, with higher rates of decline occurring during 1993–2004 and 2018–2020 (APC = -7.0%; 95% CI:-7.3, -6.6; *p* < 0.001 and − 7.4%; 95% CI:-12.0, -2.6; *p* = 0.005, respectively). Overall, between 1990 and 2020, the adult incidence of HIV decreased at an average rate of 4.2% (AAPC = -4.2%; 95% CI: -4.6, -3.8; *p* < 0.001) per year, from 3.38 cases per 1000 in 1990 to 0.94 cases per 1000 in 2020. Similarly, as shown in Fig. 1b, the incidence of HIV decreased between 1990 and 2020 (AAPC = -3.6%; 95% CI: -4.0, -3.3; *p* < 0.001). However, from 1990 to 1992, the incidence increased at a 9.3% (95% CI: 6.5, 12.2; *p* < 0.001) annual rate before beginning to fall. The period between 2018 and 2020 experienced the most significant decline over the entire study period (APC = -8.8%; 95% CI: -11.2, -6.4; *p* < 0.001).

Figure 1c and d show the trend in the prevalence of HIV among adults as well as the number of PLHIV. Between 1990 and 2001, adult prevalence increased, with the biggest rise occurring between 1990 and 1992 (APC = 23.8%; 95% CI: 18.1, 29.7; *p* < 0.001). The prevalence, on the other hand, fell between 2001 and 2020, with the greatest drop occurring between 2001 and 2008 (APC = -3.0%; 95% CI: -3.8, -2.3; *p* < 0.001). Between 1990 and 2020, the prevalence of HIV among adults grew by an average of 1.1% (95% CI: 0.6, 1.7; *p* < 0.001) per year, from 1.2 to 1.7% (Fig. 1c). PLHIV data showed an increase during the study period (AAPC = 4.4%; 95% CI: 3.9, 4.9; *p* < 0.001). However, the biggest rise occurred between 1990 and 1992 (APC = 25.1%; 95% CI: 21.6, 28.8; *p* < 0.001) (Fig. 1d).

### Trends in AIDS-related mortality in Ghana between 1990 and 2020

**Fig. 2:**
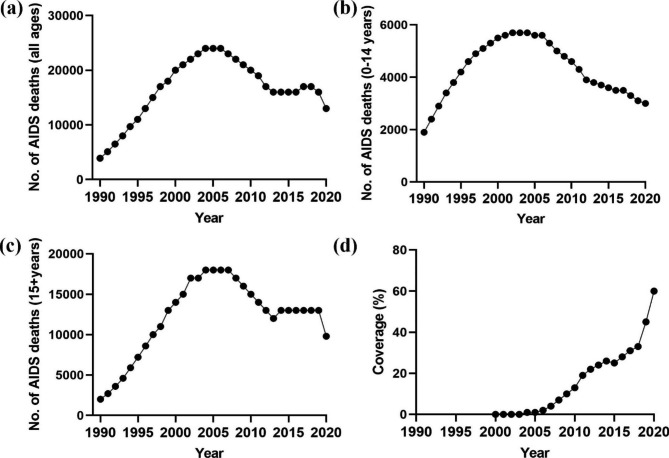
Trend of: **(a)** AIDS-related deaths (all ages); **(b)** AIDS deaths among children (0–14 years); **(c)** AIDS death among adults (15 + years); and **(d)** coverage (%) of antiretroviral therapy in Ghana between 1990 and 2020

The findings revealed that the number of deaths (among all age categories) increased during 1990–2005 and 2014–2018, with the greatest increase occurring between 1990 and 1993 (APC = 27.6%; 95% CI: 23.5, 31.8; *p* < 0.001). The number of deaths decreased from 2005 to 2014 and 2018–2020, with the most significant drop occurring between 2018 and 2020 (APC = -12.1%; 95% CI:-17.7, -6.2, *p* = 0.001), when the number of deaths fell from 17,000 to 1000 to 13,000 per 1000. Over the period 1990–2020, mortality in all age categories increased from 3,900 deaths per 1000 in 1990 to 13,000 deaths in 2020 (Fig. 2a).

During the study period, the number of AIDS-related deaths among children grew at a rate of almost 2% (95% CI: 1.0, 2.1, *p* < 0.001) each year. According to the statistics, between 1990 and 2001 there were considerable increases, with the highest rate occurring between 1990 and 1993 at 22% (95% CI: 19.0, 24.4, *p* < 0.001). The drop began in 2001, with a minimal decline between 2001 and 2006, followed by large declines between 2006 and 2020, with the greatest decline occurring between 2001 and 2012 (APC = -5.6% ; 95% CI: -6.5, -4.6, *p* < 0.001) (Fig. 2b). The trend in AIDS deaths among adults mirrored the trend among all age groups. However, from 2007 to 2013 (APC = -6.3%; 95% CI: -7.7, -4.8, *p* < 0.001) and from 2018 to 2020 (APC = -12.9; -18.7, -6.7, *p* = 0.001), there were substantial declines. During the study period, the number of fatalities among adults grew annually at a rate of 5.5% (95% CI: 4.7, 6.3, *p* < 0.001) from 2,000 deaths per 1000 in 1990 to 18,000 deaths per 1000 in 2020 (Fig. 2c).

### Trend of antiretroviral therapy coverage (ART) in Ghana from 2004 to 2020

According to the data, the population coverage of ART was 0% in 2000 until it increased to 1% in 2004. Between 2004 and 2020, coverage expanded from 1 to 60% at a 29.0% (95% CI: 24.5, 33.6; *p* < 0.001) annual rate. The most substantial increase happened between 2004 and 2010; during the defined period, coverage rose at a rate of 63.7% (95% CI: 50.9, 77.6; *p* < 0.001) each year (Fig. 2d).

### HIV and AIDS burden before and after population-wide antiretroviral therapy implementation in Ghana

**Fig. 3:**
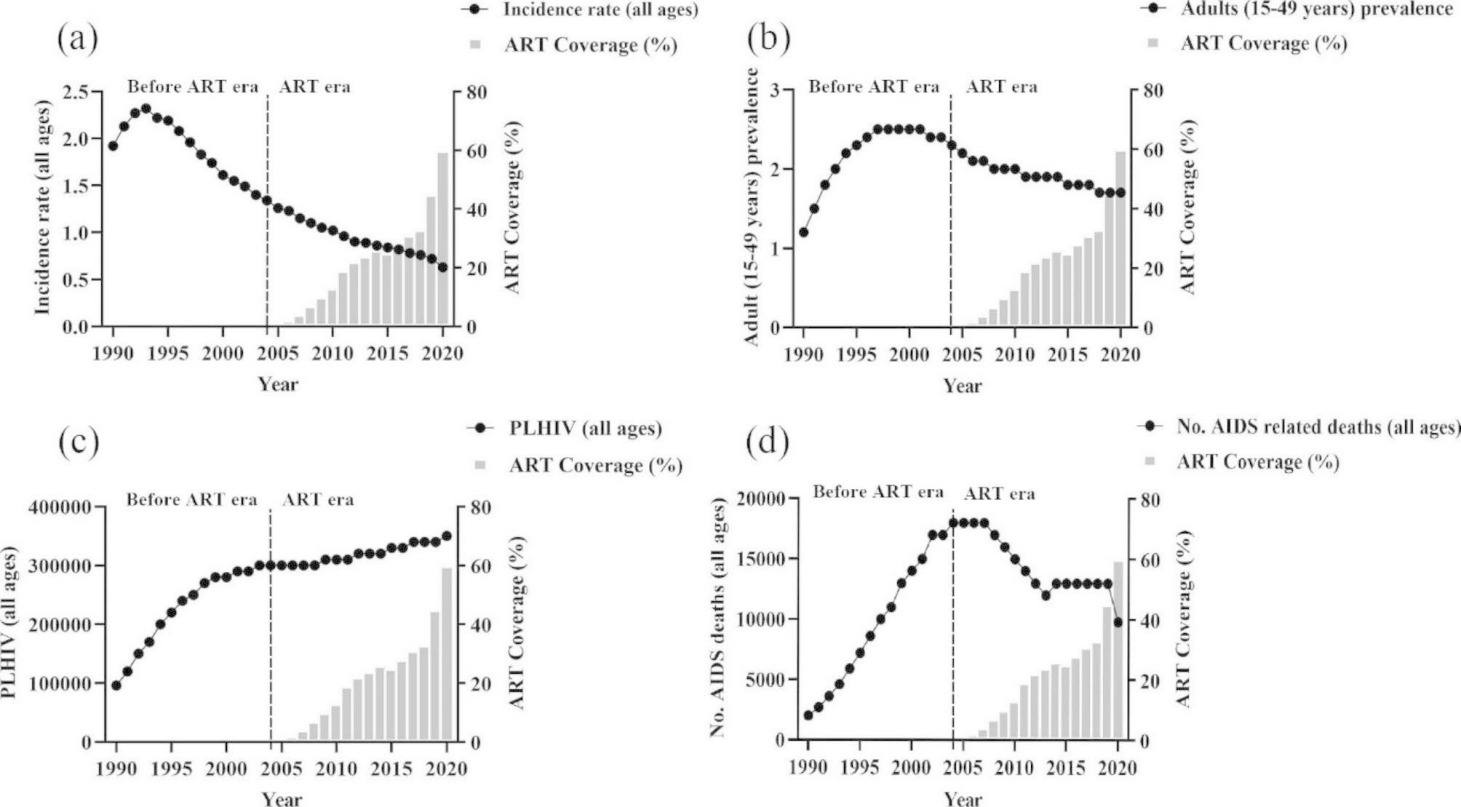
The temporal trend of: **(a)** HIV incidence rate (all ages); **(b)** adult (15–49 years) prevalence rate; **(c)** PLHIV (all ages); **(d)** Number of AIDS-related deaths (all ages) following the implementation of antiretroviral therapy. ART: antiretroviral therapy; PLHIV: People living with HIV

Figure 3; Table 2 show the trend of HIV and AIDS burden before and after the population-level ART introduction in Ghana. After the introduction of ART, the incidence, prevalence, and number of AIDS-related deaths decreased significantly compared to prior periods. For instance, after introduction, the all-age HIV incidence declined about three times as quickly (AAPC = -2.6%; 95% CI: -3.2, -1.9) between 1990 and 2004 as it did between 2004 and 2020 (AAPC = -4.5%; 95% CI: -4.9, -4.2) (Fig. 3a; Table 2). When ART was not implemented, the prevalence of HIV in adults increased by about 5% annually, whereas it decreased by about 2% per year when ART was implemented (Fig. 3b; Table 2). In contrast, the number of PLHIV increased over the entire study period. However, compared to when ART was implemented in Ghana, the increase was significantly greater when population coverage of ART was 0% (Fig. 3c; Table 2). Priore to 2004, HIV-related deaths increased at a rate of about 15%, but between 2004 and 2020, they decreased at a rate of about 4% per year (Fig. 3d; Table 2).

**Table 2:** Average annual percentage change (AAPC) in the burden of HIV/AIDS in Ghana before (1990–2004) and after antiretroviral therapy (ART) implementation (2004–2020)

Variable	Average annual percentage change (AAPC)		
	Whole period 1990–2020	Before ART 1990–2004	After ART 2004–2020
All age incidence of HIV (per 1000 uninfected population)	-3.6^d^ (-4.0, -3.3)	-2.6 ^d^ (-3.2, -1.9)	-4.5 ^d^ (-4.9, -4.2)
Adult (15–49 years) prevalence of HIV (%)	1.1 ^d^ (0.2, 2.0)	4.7 ^d^ (3.6, 5.8)	-1.9 ^d^ (-2.1, -1.6)
Number of PLHIV (all ages)	4.4 ^d^ (3.9, 4.9)	8.5 ^d^ (7.5, 9.5)	0.9 ^d^ (0.7, 1.2)
Number of AIDS deaths (all ages)	4.1 ^d^ (3.3, 5.0)	13.8 ^d^ (12.6, 15.0)	-3.6 ^d^ (-4.7, -2.5)

^d^ p < 0.05; ART: antiretroviral therapy; HIV: human immunodeficiency virus; PLHIV: people living with HIV

## Discussion

This study investigated the population-level impact of ART on the HIV/AIDS burden in Ghana between 1990 and 2020. To the best of our knowledge, this is the first attempt to examine the trend in HIV/AIDS burden in Ghana before and after the ART era in this study setting. According to the study’s findings, by the end of 2020, 60% of PLHIV were put on antiretrovirals (ARVs) that were previously once considered too expensive and complex for LMICs. The study found that between 1990 and 2020, the HIV incidence in Ghana decreased across all age groups, mirroring the global trend [3]. On the other hand, the adult prevalence, the number of PLHIV, and the number of AIDS-related deaths all increased during the same period. This is because the values in 2020 are a slightly higher than they were in 1990. Aside from the number of PLHIV, the data show that the prevalence rate and number of HIV-related deaths have decreased over the last two decades but remain higher than in 1990.

When we stratified the trends by pre- and post-ART eras, we found that when population coverage of ART was 0% (i.e. from 1990 to 2004), the HIV incidence decreased by about 3% per year, compared to about 5% per year when population coverage of ART increased from 1 to 60% (i.e. from 2004 to 2020). In terms of the HIV prevalence and the number of PLHIV, the results showed that there was a decrease in adult prevalence after ART rollout compared to the previous period. Adult HIV prevalence increased by approximately 5% per year prior to ART implementation but decreased by nearly 2% per year after implementation. The number of PLHIV, on the other hand, increased even after ART was introduced, albeit at a slower rate during ART scale-up. Only after ART became widely available did the number of AIDS-related deaths decrease at 4% per year. These results demonstrate the contribution of the public sector ART rollout in reducing the HIV/AIDS burden in Ghana.

One of the limitations of this study is that we did not take into account a number of individual and contextual factors that could have potentially confounded our findings. For instance, increased condom use worldwide since 1990 has prevented an estimated 117 million new infections between 1990 and 2019, with sub-Saharan Africa accounting for approximately 45% of the estimated infections averted [34]. As a result, we are not attributing the decline in HIV/AIDS burden in Ghana during the post-ART era solely to the rollout of ART alone; however, it is implausible that the rollout of ART has not had a significant impact on the HIV/AIDS burden in the country since its implementation. The role of ART in altering the course of HIV/AIDS has been reported in previous studies [8, 12, 35–37], providing support for the current study’s findings.

The rapid decline in HIV incidence and prevalence in Ghana subsequent to the rollout of ART can be attributed to a decrease in HIV infection in the population as a result of viral suppression and a decrease in infectivity. Evidence from epidemiological studies [6, 9] and mathematical models [4, 5] support this conclusion. Although the prevalence of HIV decreased after ART rollout, the number of PLHIV increased during the same period, albeit at a slower rate than before the introduction of ART. According to a prospective cohort study, ART increases survival time of an infected individual by suppressing HIV replication and increasing the baseline CD4 cell count [35]. According to a study conducted in southern Africa, the dramatic increase in adult HIV prevalence in Kwazulu-Natal following the introduction of ART can be explained by the increased survival of HIV-infected individuals receiving chemotherapy [37]. The improvement in life expectancy among ART recipients explains why the number of PLHIV in Ghana continues to increase despite the availability of effective treatment. However, the number has decreased since the implementation of ART. This is because ART helps reduce new HIV infections, which, along with the increase in the adult population, has contributed to the decrease in adult HIV prevalence rate observed in this study.

Prior to the availability of ART in Ghana, more children and adults were dying from AIDS-related causes. This trend was also visible in the number of AIDS-related deaths across all age groups. However, the rollout of ART correlated with a reduction in the number of AIDS-related deaths in the country. A population-based, longitudinal, multicenter study in Italy found that combined ART was effective in reducing mortality among children infected with HIV during pregnancy [10]. Other researchers also found a significant decline in HIV/AIDS-related fatalities in Africa after the introduction of ART [36, 38]. Although the introduction of ART has helped reduce the HIV/AIDS burden in Ghana, not everyone has access to therapy [3, 24]. Moreover, the prospects of HIV/AIDS-curative treatments and an effective vaccine remain uncertain.

Several limitations of the current analysis should be considered when interpreting the results. Its ecological design predisposes it to the so-called ecological fallacy. In addition, we did not adjust for individual and environmental factors that could have affected our research, as described previously in this article. As a result, we do not believe that the introduction of ART is the only reason why the HIV/AIDS burden in Ghana has decreased. Despite these limitations, our study’s findings are corroborated by other research employing rigorous statistical methodologies to quantify the impact of ART on HIV/AIDS [4, 5].

## Conclusions

The analysis found trends indicating remarkable progress in Ghana’s fight against the HIV/AIDS pandemic. Nevertheless, the HIV incidence, prevalence, and AIDS-related mortality decreased more considerably in the post-ART era, as population-level ART coverage increased from 1% to 2004 to 60% in 2020. We advocate for increased coverage of ART in Ghana.

## Electronic supplementary material

Below is the link to the electronic supplementary material.

Supplementary Material 1

## Data Availability

The data used in this study are freely available to any researcher from the UNAIDS database (https://aidsinfo.unaids.org/).

## Abbreviations

AAPC: Average annual percentage change
AIDS: Acquired immune deficiency syndrome
APC: Annual percentage change
ART: Antiretroviral therapy
ARVs: Antiretrovirals
HIV: Human immunodeficiency virus
PLHIV: People living with HIV
PMTCT: Prevention of mother-to-child transmission
STIs: sexually transmitted infections
UNAIDS: United Nations Programme on HIV/AIDS
