# Supplementary material for: Temporal trend analysis of the HIV/AIDS burden before and after the implementation of antiretroviral therapy at the population level from 1990 to 2020 in Ghana
Source: BMC Public Health. 2023 Jul 20;23:1399. doi: 10.1186/s12889-023-16321-3 (PMC10360237; doi:10.1186/s12889-023-16321-3)
Supplement: Supplementary file 1 — Supplementary Material 1 [file 12889_2023_16321_MOESM1_ESM.docx]

Table 1: Joinpoint analysis of adult (15 – 49 years) HIV incidence rate (per 1000 uninfected population) in Ghana from 1990 – 2020

| **Segment (joinpoints)** | **Lower Endpoint** | **Upper Endpoint** | **APC** | **Lower CI** | **Upper CI** | **Test Statistic (t)** | **Prob > \|t\|** |
| --- | --- | --- | --- | --- | --- | --- | --- |
| 1 | 1990 | 1993 | 4.5* | 1.9 | 7.2 | 3.6 | 0.002 |
| 2 | 1993 | 2004 | -7.0* | -7.3 | -6.6 | -38.1 | < 0.001 |
| 3 | 2004 | 2018 | -3.3* | -3.5 | -3 | -26.1 | < 0.001 |
| 4 | 2018 | 2020 | -7.4* | -12 | -2.6 | -3.2 | 0.005 |

*Indicates the annual percentage change (APC) is significantly different from zero at alpha = 0.05 level; CI: confidence interval

Table 2: Joinpoint analysis of all age incidence rate of HIV (per 1000uninfected population) in Ghana from 1990 – 2020

| **Segment** | **Lower Endpoint** | **Upper Endpoint** | **APC** | **Lower CI** | **Upper CI** | **Test Statistic (t)** | **Prob > \|t\|** |
| --- | --- | --- | --- | --- | --- | --- | --- |
| 1 | 1990 | 1992 | 9.3* | 6.50 | 12.20 | 7.3 | < 0.001 |
| 2 | 1992 | 1995 | -1.50 | -4.00 | 1.10 | -1.2 | 0.237 |
| 3 | 1995 | 2000 | -5.9* | -6.70 | -5.20 | -15.9 | < 0.001 |
| 4 | 2000 | 2012 | -4.7* | -4.90 | -4.50 | -58.7 | < 0.001 |
| 5 | 2012 | 2018 | -2.8* | -3.40 | -2.20 | -10.5 | < 0.001 |
| 6 | 2018 | 2020 | -8.8* | -11.20 | -6.40 | -7.6 | < 0.001 |

*Indicates the annual percentage change (APC) is significantly different from zero at alpha = 0.05 level; CI: confidence interval

Table 3: Joinpoint analysis of total number of people living with HIV in Ghana from 1990 – 2020

| **Segment** | **Lower Endpoint** | **Upper Endpoint** | **APC** | **Lower CI** | **Upper CI** | **Test Statistic (t)** | **Prob > \|t\|** |
| --- | --- | --- | --- | --- | --- | --- | --- |
| 1 | 1990 | 1992 | 25.1* | 21.6 | 28.8 | 16.8 | < 0.001 |
| 2 | 1992 | 1995 | 14.1* | 10.8 | 17.4 | 9.9 | < 0.001 |
| 3 | 1995 | 1998 | 6.6* | 3.6 | 9.7 | 4.8 | < 0.001 |
| 4 | 1998 | 2003 | 2.1* | 1.1 | 3 | 4.8 | < 0.001 |
| 5 | 2003 | 2007 | 0 | -1.4 | 1.4 | 0 | 0.982 |
| 6 | 2007 | 2020 | 1.2* | 1 | 1.3 | 16.4 | < 0.001 |

*Indicates the annual percentage change (APC) is significantly different from zero at alpha = 0.05 level; CI: confidence interval

Table 4: Joinpoint analysis of adult (15-49 years) prevalence rate of HIV in Ghana from 1990 – 2020

| **Segment** | **Lower Endpoint** | **Upper Endpoint** | **APC** | **Lower CI** | **Upper CI** | **Test Statistic (t)** | **Prob > \|t\|** |
| --- | --- | --- | --- | --- | --- | --- | --- |
| 1 | 1990 | 1992 | 23.8* | 18.1 | 29.7 | 9.6 | < 0.001 |
| 2 | 1992 | 1996 | 7.6* | 5.1 | 10.1 | 6.6 | < 0.001 |
| 3 | 1996 | 2001 | 0.3 | -1.2 | 1.8 | 0.4 | 0.696 |
| 4 | 2001 | 2008 | -3.0* | -3.8 | -2.3 | -8.2 | < 0.001 |
| 5 | 2008 | 2020 | -1.5* | -1.7 | -1.2 | -11.4 | < 0.001 |

*Indicates the annual percentage change (APC) is significantly different from zero at alpha = 0.05 level; CI: confidence interval

Table 5: Joinpoint analysis of AIDS-relaed deaths (all ages) in Ghana from 1990 – 2020

| **Segment** | **Lower Endpoint** | **Upper Endpoint** | **APC** | **Lower CI** | **Upper CI** | **Test Statistic (t)** | **Prob > \|t\|** |
| --- | --- | --- | --- | --- | --- | --- | --- |
| 1 | 1990 | 1993 | 27.6* | 23.5 | 31.8 | 16 | < 0.001 |
| 2 | 1993 | 1998 | 16.3* | 13.9 | 18.7 | 15.7 | < 0.001 |
| 3 | 1998 | 2005 | 5.5* | 4.4 | 6.7 | 10.5 | < 0.001 |
| 4 | 2005 | 2014 | -5.2* | -5.9 | -4.5 | -16.2 | < 0.001 |
| 5 | 2014 | 2018 | 2.4 | -0.9 | 5.8 | 1.6 | 0.139 |
| 6 | 2018 | 2020 | -12.1* | -17.7 | -6.2 | -4.2 | 0.001 |

*Indicates the annual percentage change (APC) is significantly different from zero at alpha = 0.05 level; CI: confidence interval

Table 6: Joinpoint analysis of AIDS-relaed deaths among children (0-14 years) in Ghana from 1990 – 2020

| **Segment** | **Lower Endpoint** | **Upper Endpoint** | **APC** | **Lower CI** | **Upper CI** | **Test Statistic (t)** | **Prob > \|t\|** |
| --- | --- | --- | --- | --- | --- | --- | --- |
| 1 | 1990 | 1993 | 21.6* | 19.0 | 24.4 | 18.9 | < 0.001 |
| 2 | 1993 | 1997 | 9.2* | 6.8 | 11.7 | 8.5 | < 0.001 |
| 3 | 1997 | 2001 | 3.4* | 1.1 | 5.7 | 3.2 | 0.006 |
| 4 | 2001 | 2006 | -0.1 | -1.5 | 1.3 | -0.1 | 0.915 |
| 5 | 2006 | 2012 | -5.6* | -6.5 | -4.6 | -12.4 | < 0.001 |
| 6 | 2012 | 2020 | -3.3* | -3.8 | -2.9 | -15.0 | < 0.001 |

*Indicates the annual percentage change (APC) is significantly different from zero at alpha = 0.05 level; CI: confidence interval

Table 7: Table 6: Joinpoint analysis of AIDS-relaed deaths among adults (15+ years) in Ghana from 1990 – 2020

| **Segment** | **Lower Endpoint** | **Upper Endpoint** | **APC** | **Lower CI** | **Upper CI** | **Test Statistic (t)** | **Prob > \|t\|** |
| --- | --- | --- | --- | --- | --- | --- | --- |
| 1 | 1990 | 1995 | 30.2* | 28.2 | 32.2 | 37.0 | < 0.001 |
| 2 | 1995 | 2002 | 12.3* | 11.0 | 13.6 | 21.6 | < 0.001 |
| 3 | 2002 | 2007 | 0.9 | -1.3 | 3.1 | 0.9 | 0.397 |
| 4 | 2007 | 2013 | -6.3* | -7.7 | -4.8 | -9.1 | < 0.001 |
| 5 | 2013 | 2018 | 1.9 | -0.2 | 4.2 | 1.9 | 0.077 |
| 6 | 2018 | 2020 | -12.9* | -18.7 | -6.7 | -4.3 | 0.001 |

*Indicates the annual percentage change (APC) is significantly different from zero at alpha = 0.05 level; CI: confidence interval
